# Real-world effectiveness of long-acting injectable antipsychotic treatments in a nationwide cohort of 12,373 patients with schizophrenia-spectrum disorders

**DOI:** 10.1038/s41380-023-02175-z

**Published:** 2023-07-21

**Authors:** Laurent Boyer, Bruno Falissard, Philippe Nuss, Cedric Collin, Stephanie Duret, Marc Rabbani, Isabelle De Chefdebien, Isabelle Tonelli, Pierre Michel Llorca, Guillaume Fond

**Affiliations:** 1 Centre for Studies and Research on Health Services and Quality of Life (CEReSS), Aix-Marseille University, Marseille, France; 2 Universite Paris-Saclay, UVSQ, Inserm, Developmental Psychiatry, CESP, Villejuif, France; 3 AP-HP, Service de Psychiatrie et de Psychologie Médicale, Paris, France; 4 IQVIA Operations, Paris, France; 5 Lundbeck SAS, Puteaux, Paris, France; 6 Otsuka Pharmaceutical, Paris, France; 7 University of Clermont Auvergne, Clermont-Ferrand, France

**Keywords:** Psychology, Neuroscience

## Abstract

This mirror-image study aimed to evaluate the real-life effectiveness of long-acting injectable antipsychotics (LAI) in schizophrenia. Patients with schizophrenia initiating LAIs January 2015–December 2016 were enrolled from the French National Health Data System (SNDS). Standardized mean differences (SMD > 0.1 deemed clinically significant) were calculated for psychiatric healthcare resource utilization measures assessed one year before (during oral AP treatment) and one year after LAI initiation. LAI effectiveness was analyzed overall and by age group, gender and compliance to oral AP, defined as exposure to an AP for at least 80% of the year before LAI initiation. 12,373 patients were included. LAIs were more frequently initiated in men (58.1%), young (18–34 years, 42.0%) and non-compliant (63.7%) patients. LAI initiation was effective in reducing the number and duration of psychiatric hospitalizations and psychiatric emergency department (ED) admissions in non-compliant patients (SMD = −0.19, −0.26 and −0.12, respectively), but not in compliant patients. First-generation LAIs, paliperidone and aripiprazole LAIs reduced psychiatric hospitalizations (SMD = −0.20, −0.24, −0.21, respectively) and ED admissions (SMD = −0.15, −0.13, −0.15, respectively). No differences in effectiveness were found for age or gender. In compliant patients, only aripiprazole LAI reduced the number of psychiatric hospitalizations (SMD = -0.13). Risperidone and paliperidone LAIs increased hospitalization duration (SMD = 0.15 and 0.18, respectively). The prescription of LAIs (except risperidone) should be recommended in all non-compliant patients, even in women and patients aged 35 or older. The lower frequency of administration of LAIs than of oral APs may improve compliance and hence reduce the risk of relapse. Aripiprazole LAI may represent a treatment of choice for compliant patients that should be further investigated.

## Introduction

Schizophrenia is a complex psychiatric disorder, with a global prevalence of 0.32% in 2022 [1]. Despite this relatively low prevalence, schizophrenia is associated with a substantial disease burden arising from a chronic course characterized by acute phases and periods of residual symptoms together with a high frequency of underlying physical comorbidities and an excess risk of suicide. Collectively, these factors contribute to high rates of morbidity and mortality among patients with schizophrenia. A challenge of schizophrenia from a clinical and pharmacoeconomic perspective is to reduce psychiatric hospitalizations and emergency department (ED) admissions, which are indicative of uncontrolled disease.

Introduction of the first-generation antipsychotics (APs) in the 1950s led to a marked decrease in hospitalizations in patients with schizophrenia and marked the beginning of ambulatory care. However, a large proportion of patients had poor compliance with treatment due to lack of insight or poor tolerance resulting in treatment withdrawal and psychotic relapse. Long-acting injectable APs (LAIs) were therefore developed between the 1970s and the 1980s to improve compliance, but evidence of effectiveness was limited due to the lack of large-scale studies at the time. Introduced in the 1990s, clozapine and other second-generation APs demonstrated better effectiveness than first-generation APs [2]. Risperidone was the first second-generation LAI to become available in France (in 2004). The approval of these products was based on the results of randomized controlled trials showing the effectiveness of LAIs in reducing psychotic symptomatology and relapse risk compared to oral formulations [3, 4]. However, these studies were criticized as they were industry-sponsored (before current GxP standards) and due to their selection bias (i.e., selection of patients with good compliance and without severe addictions or suicide risk) and potential loss to follow-up [5]. With the advantage of allowing within-patient comparisons, mirror-image studies have demonstrated the effectiveness of LAIs in real-life scenarios for patients with schizophrenia using proxy measures, such as healthcare resource utilization [6–9]. Moreover, a meta-analysis of 25 mirror-image studies encompassing 5940 patients with schizophrenia from 28 countries showed superiority of LAIs over oral APs in preventing hospitalizations and reducing hospitalization rates, in contrast to findings from a meta-analysis of randomized controlled trials [10]. However, mirror-image studies have generally enrolled small study populations (46–850 patients) from a single center or database [6–9].

Nationwide cohort studies carried out in Finland and Sweden have tried to address these limitations and have shown that LAIs enhance compliance and improve clinical outcomes and quality of life in patients with schizophrenia [11–14]. As a consequence, many guidelines advocate LAIs for improving adherence to AP medication [15, 16]. These recommendations are challenged by a persistent medication non-adherence rate of approximately 50% attested by recent studies [17, 18], leading to an increased risk of relapse and hospitalization with consequent increases in healthcare resource utilization and costs, and adverse effects on patient quality of life [6, 19, 20]. One example of the impact of non-adherence on resource utilization is in ED admissions [21]. Therefore, reducing ED admissions is a priority to reduce the burden of schizophrenia for the patient, her/his relatives and for the healthcare system.

In addition, it is unclear if LAIs should be prescribed to all patients or for specific subgroups, such as male, younger, and/or non-compliant patients, who may benefit more from LAIs due to higher risk of psychotic relapse [22, 23]. It has been argued that clinicians’ over-estimation of good compliance of their own patients was a barrier to the more widespread use of LAIs [22, 23], but it is not clear if the advantages of LAIs only apply in patients with poor compliance. More recently introduced second-generation LAIs (paliperidone and aripiprazole) may provide benefits in a range of patient subgroups.

We present results from a study combining the advantages of a population-based database and a mirror-image design (to our knowledge, the first population-based study of its kind). This study aimed to evaluate the real-life effectiveness of first- and second-generation LAIs in reducing psychiatric hospitalizations and ED admissions in patients with schizophrenia. Our secondary objectives were to determine whether LAIs are more effective than oral forms in all age classes, in both genders and in non-compliant and compliant patients.

We hypothesized that second-generation LAIs would out-perform oral APs in reducing psychiatric hospitalizations and ED admissions, especially in non-compliant patients.

## Methods

### Study design, data sources and population

This was a nationwide mirror-image study of patients with schizophrenia treated with LAIs enrolled in the French National Health Data System (SNDS) between 1 January 2014 and 31 December 2017. Within this study duration, a mirror-image analysis was performed to compare endpoints during a minimum follow-up period of one year before and one year after LAI initiation (1 January 2015 to 31 December 2016) using matched data for each patient (i.e., each individual acted as their own control). The SNDS contains comprehensive, individualized and anonymized data on health spending reimbursements for most individuals living in France [24]. The SNDS includes beneficiary data on sociodemographic, medical, hospital admissions and treatment delivery data from the French Health Insurance Database (SNIIRAM) and the *Programme pour la Médicalisation du Système d’Information*/Program for the Medicalization of the Information System (PMSI) [24–26].

#### Inclusion criteria

Eligible patients were identified in a four-stage selection process as the identification of patients solely based on treatment is not sufficient to distinguish between schizophrenia and other conditions for which APs may have been administered. In the initial selection stage, patients with schizophrenia were identified based on the criteria of an active long-term disorder during the study with an international classification of diseases – 10th revision (ICD-10) diagnostic code of F2.X for schizophrenia (and related disorders) (Table S1); and/or at least one hospital stay in the Medical/Surgical/Gynaecological-Obstetrics (MCO; subsequently referred to as non-psychiatric hospitalization) sector with a main diagnosis (MD) or related diagnosis indicative of schizophrenia; and/or at least one hospital stay with an MD or associated diagnosis indicative of schizophrenia in the Psychiatric Hospital Discharge Database (RIM-P; classified as part-time, full-time or outpatient status); and at least 3 deliveries (on different dates; between 2012 and 2017) of Anatomical Therapeutic Chemical/ATC class N05A ‘antipsychotic agents’ (except N05AN/lithium). In the second stage, patients with schizophrenia identified at initial selection were eligible for inclusion in the study if they had started LAI therapy between 1 January 2015 and 31 December 2016 (to allow assessment of the patient in the year preceding starting LAI therapy and at least one year follow-up). Initiation of a LAI treatment was specified by no reimbursement for any LAI in the 3 years preceding the delivery of the given LAI. The index date was defined as the first date on which the LAI was dispensed. The third stage involved selection of patients with schizophrenia who were alive and with ≥1 year of data before and after the index date. Only patients aged ≥18 years were included in the final selection stage.

#### Exclusion criteria

Patients who were under 18 years of age at the index date and insufficiently recognizable as an individual (e.g., fictional ‘PseudoNIR’ individuals, twins) were ineligible for the study. Patients were also excluded if they were not enrolled in the general scheme or a local mutual association, or had received treatments reimbursed in Mayotte.

### Psychiatric healthcare resource utilization

Proxy healthcare resource utilization measures of effectiveness were assessed for each patient one year prior to and one year after initiation of an LAI (index date). These endpoints were the number and duration of psychiatric hospitalizations (full- or part-time stays in psychiatric hospitals or in general hospitals for psychiatric reasons), and the number of psychiatric ED admissions.

### Data collection

Data relating to sociodemographic characteristics (age classes: 18–34, 35–49 and ≥50 years; gender; low socio-economic level (defined by the additional French Universal Health Cover (*Couverture maladie universelle*/CMU) that is provided for the poorest 10% of households in France [27]); comorbidities (smoking, alcohol, or substance addiction, Charlson Comorbidity score: 0, 1–2, ≥3); characteristics of hospital stay (origin of the patient: home, transfer from other hospital, emergency ward); category of care; and initiation of LAIs were extracted from the SNDS database.

### Compliance definition

Compliance was defined as exposure to an oral AP for at least 80% of the year before LAI initiation, according to the data collected from the SNDS. If exposed for less than 80% of the year, the patient is classified as non-compliant. This definition aligns with recommended expert consensus guidance that endorsed 80% or more of medication taken as a threshold for compliance [28].

### Statistical analyses

Effectiveness analyses were performed in the overall patient population and in pre-specified subgroups (age groups [18–34, 35–49 and ≥50 years], gender and compliant/non-compliant).

Parametric (paired *T* test) or non-parametric (Wilcoxon signed rank test) analyses were undertaken for comparisons of continuous variables, while McNemar’s Chi-square tests were performed for categorical data. The tests were two-tailed with a significance level of 5%. Standardized mean differences (SMD) were calculated to identify meaningful differences between time periods, with an SMD > 0.1 deemed clinically significant (i.e., equivalent to a phi coefficient of 0.05 and negligible correlation) [29]. No formal sample size estimation was necessary, but with approximately 86% of the adult French population included in the SNDS, a sample size of between 400,00 and 450,000 was expected. In 2017, the proportion of incident LAI users among the French population in the SNDS was estimated to be between 31,400 and 42,000 (for all indications including schizophrenia and bipolar disorders). The four-stage selection process for eligibility described above was applied to this population of incident LAI users to arrive at a finalized sample.

Analyses were performed using the software SAS® Enterprise Guide v7.4 (SAS Institute North Carolina, USA).

## Results

### Patient characteristics

**Fig. 1:**
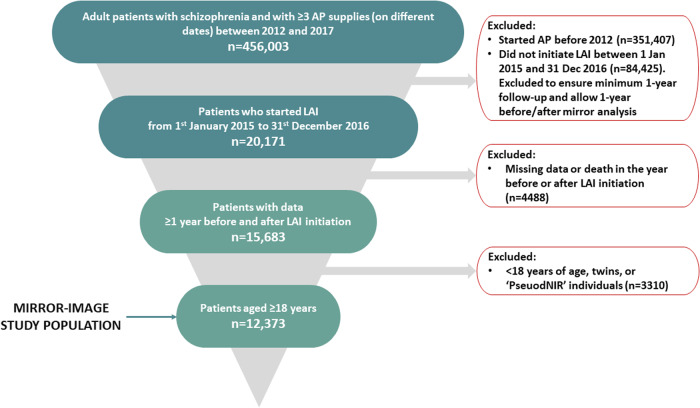
Study flow diagram. Each panel describe the selection process.

Among the selected population of 456,003 patients with schizophrenia who were AP users, a total of 12,373 were included in the study after application of all selection criteria (Fig. 1). Of these, 3853 (31.1%) initiated aripiprazole LAI, 3582 (29.0%) paliperidone LAI, 3144 (25.4%) first-generation LAIs and 1794 (14.5%) long-acting risperidone. Characteristics of the overall study population and by compliance status are shown in Table 1.

**Table 1:** Characteristics of patients with schizophrenia treated with LAIs (overall population and by compliance status).

	Total population (*N* = 12,373)	Compliant patients (*N* = 4496; 36.4%)	Non-compliant patients (*N* = 7877; 63.7%)	*P*-Value
	*n* (%)	*n* (%)	*n* (%)	
Age groups (years)				<0.001
18–34	5200 (42.0)	1593 (35.4)	3607 (45.8)	
35–49	4661 (37.7)	1832 (40.7)	2829 (35.9)	
≥50	2512 (20.3)	1071 (23.8)	1441 (18.3)	
Gender				<0.001
Male	7187 (58.1)	2471 (55.0)	4716 (59.9)	
Female	5186 (41.9)	2025 (45.0)	3161 (41.1)	
Beneficiary of an additional CMU	3388 (27.4)	968 (21.5)	2420 (30.7)	<0.001
Initiated LAI				<0.001
1G	3144 (25.4)	1194 (26.6)	1950 (24.8)	
2G				
Aripiprazole	3853 (31.1)	1550 (34.5)	2303 (29.2)	
Paliperidone	3582 (29.0)	1122 (25.0)	2460 (31.2)	
Risperidone	1794 (14.5)	630 (14.0)	1164 (14.8)	

*1G* first-generation, *2G* second-generation, *LAI* long-acting injectable antipsychotic, *CMU* French Universal Health Cover.

Overall, 28% of LAI initiations were undertaken during psychiatric hospitalization. Most patients initiated LAIs when they were not compliant with their previous AP treatment (7877 [63.7%] non-compliant [<80% exposure to an oral AP in the year before LAI initiation] versus 4496 [36.4%] compliant). Male gender, age <50 years and a lower socio-economic status were numerically more frequent in the non-compliant group (Table 1).

### Effectiveness of LAIs in reducing healthcare utilization

**Fig. 2:**
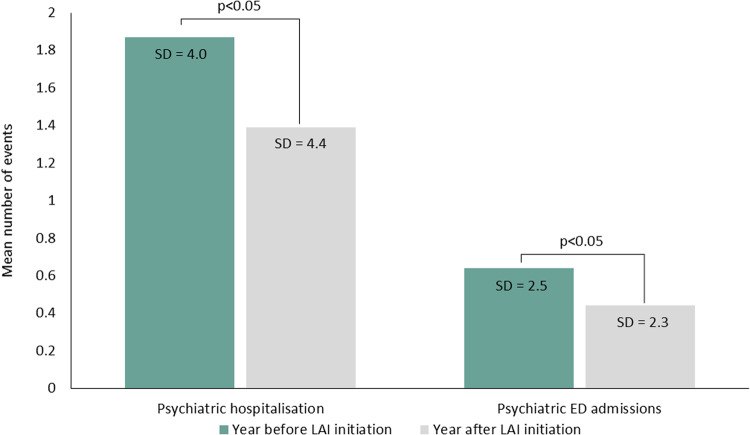
Healthcare utilization relating to psychiatric care one year before LAI initiation (during oral AP treatment) and one year after LAI initiation.

Table 2 and Fig. 2 summarize the SMDs for effectiveness endpoints measured during the year before (during oral AP treatment) and during the year after LAI initiation. The mean number and duration of psychiatric hospitalizations significantly declined in the year after initiating an LAI, but not the mean number of psychiatric ED admissions (Table 2; Fig. 2).

**Table 2:** SMD for effectiveness endpoints measured one year before LAI initiation (during oral AP treatment) and one year after LAI initiation in overall patients with schizophrenia and by compliance status.

	Total population (*N* = 12,373)	Compliant patients (*n* = 4496)	Non-compliant patients (*n* = 7877)
	SMD (95 CI%)	SMD (95 CI%)	SMD (95 CI%)
Number of psychiatric hospitalizations*	**−0.16** (−0.18; −0.13)	−0.07 (−0.10; −0.02)	**−0.19** (−0.23; −0.17)
Duration of psychiatric hospitalizations*	**−0.15** (−0.18; −0.13)	0.07 (0.02; 0.11)	**−0.26** (−0.29; −0.23)
Number of psychiatric ED admissions*	−0.09 (−0.12; −0.07)	−0.07 (−0.11; −0.03)	**−0.12** (−0.15; −0.9)

SMDs deemed clinically significant (>0.1) are highlighted in bold.

*AP* antipsychotic, *LAI* long-acting injectable antipsychotic, *ED* emergency department, *SMD* standardized mean difference.

*Statistically significant differences (*p* < 0.05) in the total sample, compliant and non-compliant patients.

### Subgroup analyses: non-compliant and compliant groups

Initiation of LAIs in the non-compliant group showed superior effectiveness compared with the compliant group (Table 2). In non-compliant patients, LAI initiation was associated with significant decreases in the number (2.01 [SD 4.3] pre- and 1.35 [4.8] post-initiation; SMD = −0.19) and duration (52.13 [80.0] days pre- and 35.87 [84.9] days post-initiation; SMD = −0.26) of psychiatric hospitalizations and psychiatric ED admissions (0.18 [0.7] pre- and 0.11 [1.1] post-initiation; SMD = −0.12) versus the 1-year period prior to initiation.

As shown in Table 3, similar results were observed for each individual LAI in non-compliant patients, with significant decreases in the number of psychiatric hospitalizations and psychiatric ED admissions with first-generation LAIs, paliperidone LAI, aripiprazole LAI and risperidone LAI compared with the 1-year period prior to initiation. There were also decreases in the mean duration of psychiatric hospitalizations that reached significance following initiation of first-generation LAIs, paliperidone and aripiprazole, but not following risperidone initiation.

**Table 3:** SMD for effectiveness endpoints by LAI in compliant and non-compliant patients with schizophrenia.

	Paliperidone (*n* = 3582)	Aripiprazole (*n* = 3853)	Risperidone (*n* = 1794)	LAI 1 G (*n* = 3144)
	SMD (95% CI)	SMD (95% CI)	SMD (95% CI)	SMD (95% CI)
Non-compliant patients	***n*** = **2460**	***n*** = **2303**	***n*** = **1164**	***n*** = **1950**
Number of psychiatric hospitalizations	**−0.24** (−0.29; −0.18)	**−0.21** (−0.27; −0.15)	**−0.23** (−0.31; −0.15)	**−0.20** (−0.26; −0.13)
Duration of psychiatric hospitalizations	**−0.30** (−0.35; −0.24)	**−0.27** (−0.33; −0.21)	−0.08 (−0.16; 0.00)	**−0.31** (−0.38; −0.25)
Number of psychiatric ED admissions	**−0.13** (−0.19; −0.07)	**−0.15** (−0.20; −0.09)	**−0.16** (−0.24; −0.07)	**−0.15** (−0.21; −0.09)
Compliant patients	***n*** = **1122**	***n*** = **1550**	***n*** = **630**	***n*** = **1194**
Number of psychiatric hospitalizations	−0.01 (−0.09; 0.07)	**−0.13** (−0.2; −0.06)	−0.02 (−0.13; −0.09)	−0.08 (−0.16; 0.01)
Duration of psychiatric hospitalizations	**0.17** (0.08; 0.25)	0.04 (−0.0; 0.11)	**0.15** (0.04; 0.26)	−0.03 (−0.11; −0.05)
Number of psychiatric ED admissions	−0.04 (−0.12; 0.05)	−0.02 (−0.09; 0.05)	0.05 (−0.06; 0.16)	−0.05 (−0.13; 0.03)

SMDs deemed clinically significant (>0.1) are highlighted in bold.

*1G* first-generation, *LAI* long-acting injectable antipsychotic, *ED* emergency department, *SMD* standardized mean difference.

In the compliant patient group, aripiprazole was the only LAI associated with a clinically significant reduction in the number of psychiatric hospitalizations (Table 3). Increased durations of psychiatric hospitalizations were observed following initiation of risperidone and paliperidone. Commencement in compliant patients was not followed by clinically significantly reduced psychiatric ED admissions with any individual LAI.

### Subgroup analyses: age groups and gender

Overall, reductions in psychiatric hospitalizations and psychiatric ED admissions were demonstrated following LAI initiation regardless of age group or gender (Table S2).

## Discussion

To our knowledge, this is the first population-based study evaluating the real-life effectiveness of LAIs among patients with schizophrenia-spectrum disorders with a mirror-image design. The results show that LAIs were more frequently prescribed to young, male, and non-compliant patients, and that a broader range of patients can benefit from LAIs (except risperidone) in reducing healthcare utilization, particularly non-compliant patients, including female and older patients, which is a significant new finding that could change clinical practice. Aripiprazole LAI was also found to reduce the number of psychiatric hospitalizations in compliant patients, which needs verification in other studies. Strikingly, risperidone and paliperidone LAI were associated with increased psychiatric hospitalization durations in compliant patients.

When compared with the 1-year period before initiation that included treatment with oral APs, initiation of LAIs reduced the rate and duration of psychiatric hospitalizations by 20–26% along with a 29% decrease in the number of psychiatric ED admissions (see Fig. 2). These results are in line with findings from previous single-center mirror-image studies showing superior improvement in rates (54–89%) and length of psychiatric or overall hospitalization (49–88%) when analyzing the effects of switching from oral APs to LAIs (first-generation LAIs, risperidone, olanzapine, aripiprazole and/or paliperidone) over follow-up periods of up to 2 years in clinical practice in Europe (Croatia, Italy, Germany, UK) and Asia (China, Korea) [6–9, 30, 31], as well as a meta-analysis of 25 mirror-image studies highlighting a 62% risk reduction in hospitalization rate with LAIs versus oral APs [10]. Additionally, other nationwide studies conducted in Finland, Sweden, Korea and the USA have also reported benefits of first-generation or second-generation (olanzapine, risperidone or paliperidone) LAIs on healthcare resource utilization outcomes, which reported 22–64% lower risks of rehospitalization with LAIs than with oral APs [17, 32, 33]. It should be noted that most of these studies (except [6, 8, 30]) were conducted in an earlier dataset (pre-2013) that predated aripiprazole LAI approval. Overall, nationwide studies seem to report lower effectiveness of LAI initiation compared with clinical trials, probably due to less stringent selection criteria. However, LAIs remain effective in reducing psychiatric acute care utilization, which is a priority to reduce the burden of schizophrenia for the patients, their relatives and for the healthcare professionals.

Our analysis showed that the effects of overall and individual LAIs were markedly greater in magnitude in non-compliant than compliant patients. Compared with the 1-year pre-initiation period, LAIs reduced subsequent psychiatric acute care indicators (hospitalization rates and number of ED admissions) by 33–39% in non-compliant patients who formed the majority of the study population. In our study, LAIs were most commonly initiated in non-compliant patients who were more frequently male, younger than 50 years of age and of low socio-economic status. These patient characteristics are in line with those identified in most studied schizophrenia populations where non-compliance rates are usually high [20]. Furthermore, our subgroup analyses demonstrated the effectiveness of LAIs regardless of age group or gender. Although studies have evaluated the effects of LAIs on compliance in patients with schizophrenia [32], few have analyzed LAI effects on healthcare resource use by compliance status [8] and none have taken confounders, such as age and gender into account. However, while not evaluating compliance per se, nationwide studies have highlighted 5–79% lower risks of rehospitalization for LAI use versus no use [17, 32, 33], coherent with our observed benefits of LAIs on hospitalization outcomes in non-compliant patients. A caveat to these results is that the high non-compliance rate in the year preceding LAI initiation could include patients who started LAIs within a few months of initiating oral AP therapy and who therefore would meet the criteria of ‘exposure to an oral AP for <80% of the year before LAI initiation’.

Among compliant patients, only aripiprazole LAI reduced psychiatric hospitalization frequency (by 20%), compared with oral APs, while paliperidone and risperidone LAIs were associated with an approximately 25% longer hospital stay compared with oral APs in this subgroup. We lack study data exploring the compliance status of patients to discuss these results. As non-compliant patients are more frequent than compliant ones, the results of unselected previous studies may be mostly explained by the predominance of non-compliant patients [7, 8, 32, 33]. However, a mirror-image nationwide study conducted in New Zealand reported 43% increased hospital stays 12 months post-risperidone LAI initiation, consistent with the present results in compliant patients [34]. The frequency of LAI administration (i.e., monthly versus every 2 weeks) may represent an important factor in reducing psychiatric hospitalization stays [35]. More specifically, prolonged hospital stays following risperidone and paliperidone LAI initiations among compliant patients may reflect the fact that these antipsychotics require a 3-week oral supplementation (for risperidone) or two initial injections one week apart (for paliperidone) as opposed to haloperidol, which requires only one initial injection. However, aripiprazole also requires a 14-day oral supplement upon initiation and this agent was not associated with prolonged hospital stays. Published data on the real-life effectiveness of aripiprazole LAI have only recently been published [36]. Three small mirror-image studies have reported findings for aripiprazole LAI that are in agreement with our findings [8, 37, 38]. For example, one study evaluated 148 UK patients (65% with schizophrenia) receiving aripiprazole LAI between 2014 and 2018 and reported >79% reductions in hospitalization number and duration in both fully compliant and partially compliant (≥50% of injections) patients in the 2-year period following LAI initiation [8]. It may be speculated that the reported favorable tolerability profile of aripiprazole [39] may underlie the reduction in admission rates among compliant patients with schizophrenia. Furthermore, a potentially reduced propensity for dopamine supersensitivity psychosis with long-term aripiprazole treatment has been implied as an underlying reason for the observed reduction in hospitalizations regardless of compliance status [8]. In this mirror-image study, each patient served as her/his own control, which is a strength compared to other nationwide studies in which aripiprazole may be more frequently prescribed to less severely ill patients (with fewer positive symptoms or aggressiveness) [8]. Overall, our observations emphasize the importance of compliance to oral APs for beneficial patient outcomes, but also suggest that some second-generation LAIs may offer benefits even when an individual is compliant with their oral AP regimen. To further investigate healthcare utilization and indirect costs, a future study could use a medico-economic approach to assess differences between oral APs and LAIs. Another area of future research would be to assess the impact of LAI dose on compliance and/or relapse and whether this could allow the development of lower dose LAI strategies in future.

This study reflects the prescribing habits of French psychiatrists and highlights the profile of LAI use according to their clinical judgment in a large population of patients with schizophrenia. In particular, the findings indicate their use of LAIs in a population of individuals meeting non-compliance criteria. Our results suggest that non-compliant women and non-compliant patients aged 35 years or older may be under prescribed LAIs; as these patients potentially benefit from LAIs, these results may encourage clinicians to prescribe more to these populations. These findings are important in providing complementary data to those of randomized controlled trials [40].

Study limitations, in addition to those discussed above, were inherent to the mirror-image and claims-based design, such as potential mis-coding errors that may affect prevalence, the exclusion of some agents such as olanzapine LAI because it is used exclusively for hospital use in France, and the potential for confounding due to the absence of a control group and observational nature of the study, as well as potential biases arising from the natural course of the illness and time effects [6, 7, 10]. Furthermore, patients treated with clozapine could have been analyzed separately, in order to identify patients with the most severe/resistant schizophrenia, but this was not done in the current study. This could be an important addition to future studies as a proxy for schizophrenia resistance/severity. Another limitation was that the different types of schizophrenia-spectrum disorders (e.g., schizoaffective disorders and recurrent delusional disorders) were not subjected to subgroup analyses, which could be a useful addition for future studies. Patients with substance use disorders were not separately assessed in this analysis, and therefore, we cannot comment on the response profile of these individuals – this may be a useful addition to future analyses. As this study was not a formal head-to-head comparison, conclusions could not be reached with respect to differences between individual LAIs. Although caution should be exercised when extrapolating results from this French population to other countries, the validity of the study findings is underscored by the fact that patients were drawn from a nationwide population (i.e., without selection bias) and were analyzed according to a pre-specified protocol. LAIs are frequently prescribed when patients are involuntarily committed to treatment by court order [41]. Within the framework of involuntary commitment, the patient must take her/his treatment and attend her/his follow-up appointments, without which he/she may be quickly re-hospitalized. It is therefore difficult to distinguish whether improvements in healthcare utilization following LAI initiation are due to LAIs or as a result of increased involuntary commitment. Nevertheless, our results lend support to previous findings in providing a rationale for switching to LAIs in patients with poor compliance to oral APs and show effectiveness of aripiprazole LAI in compliant patients. A further aspect of the trial that is open to discussion is the use of 80% exposure to an oral AP as the cut-off to define compliance. Some patients, for example those who had experienced one or more long-term hospitalizations, may have been wrongly classified as non-compliant despite using their oral AP during their stay. However, we do not believe that this limitation would have any impact on the results of the study, since an individual wrongly classified as non-compliant would tend to decrease the difference observed before and after treatment.

In conclusion, this study, drawing from a nationwide population, highlights the real-life effectiveness of LAIs in improving outcomes among non-compliant patients with schizophrenia regardless of age and gender. While LAIs are more frequently prescribed to young, male and non-compliant patients, female and older patients could also greatly benefit from them. This finding is potentially practice changing. In the choice of AP treatment, assessing compliance is crucial to optimal outcomes, regardless of the patient’s age or gender. Poor compliance to medication regimens is a common problem that can compromise treatment efficacy and patient outcomes [17, 18]. To address this issue, clinicians should consider proposing LAIs whenever available for patients with poor compliance. By doing so, clinicians can ensure that patients receive continuous treatment, which can improve overall treatment outcomes and reduce the risk of relapse. Aripiprazole LAI was also effective in reducing psychiatric-related hospitalizations among compliant patients. In today’s healthcare landscape, where patient-centered care and quality outcomes are increasingly important, assessing and addressing medication compliance is more critical than ever. Given the potential benefits of LAIs, action may be needed to raise awareness among healthcare providers, patients and their carers, update guidelines, and to determine when LAI treatment should be optimally initiated [23]. Further replication of these findings in a separate patient cohort using the relatively stringent compliance definition employed in this study may be warranted, with delineation of comparisons between varying oral AP regimens.

### Supplementary information

Supplementary materials
