# Supplementary material for: Real-world effectiveness of long-acting injectable antipsychotic treatments in a nationwide cohort of 12,373 patients with schizophrenia-spectrum disorders
Source: Mol Psychiatry. 2023 Jul 21;28(9):3709–16. doi: 10.1038/s41380-023-02175-z (PMC10730399; doi:10.1038/s41380-023-02175-z)
Supplement: Supplementary file 1 — Supplementary materials [file 41380_2023_2175_MOESM1_ESM.docx]

**Supplementary materials**

Table S1. ICD-10 diagnostic codes for schizophrenia

|  | **Code**  **ICD-10** | **Description** |
| --- | --- | --- |
| Schizophrenia | F20.0 | Paranoid schizophrenia |
|  | F20.1 | Hebephrenic schizophrenia |
|  | F20.2 | Catatonic schizophrenia |
|  | F20.3 | Undifferentiated schizophrenia |
|  | F20.4 | Post-schizophrenic depression |
|  | F20.5 | Residual schizophrenia |
|  | F20.6 | Simple schizophrenia |
|  | F20.8 | Other schizophrenia |
|  | F20.9 | Schizophrenia, unspecified |
|  | F20 | Schizophrenia |
|  | F21 | Schizotypal disorder |
|  | F22 | Persistent delusional disorders |
|  | F23 | Acute and transient psychotic disorders |
|  | F24 | Induced delusional disorder |
|  | F25 | Schizoaffective disorders |
|  | F28 | Other nonorganic psychotic disorders |
|  | F29 | Unspecified nonorganic psychosis |

ICD-10, International Classification of Diseases – 10^th^ revision.

Table S2. Effectiveness endpoints measured one year before LAI initiation (during oral AP treatment) and one year post LAI initiation in patients with schizophrenia by age group and gender

|  | **Age groups (years)** | | | **Gender** | |
| --- | --- | --- | --- | --- | --- |
|  | **18–34  (n=5200)** | **35–49  (n=4661)** | **≥50  (n=2512)** | **Male**  **(n=7187)** | **Female  (n=5286)** |
|  | **Mean  (SD)** | **Mean (SD)** | **Mean  (SD)** | **Mean (SD)** | **Mean (SD)** |
| Number of psychiatric hospitalizations*  1-year pre-initiation  1-year post-initiation | 1.81 (3.3)  1.35 (3.6) | 1.88 (3.6)  1.32 (4.3) | 2.00 (5.6)  1.59 (5.9) | 1.82 (3.6)  1.32 (3.6) | 1.95 (4.5)  1.49 (5.3) |
| Duration of psychiatric hospitalizations*  1-year pre-initiation  1-year post-initiation | 45.43 (76.3)  37.10 (85.2) | 47.40 (80.3)  37.51 (86.8) | 43.29 (72.6)  34.28 (80.8) | 47.00 (79.1)  37.94 (86.9) | 43.98 (74.3)  34.94 (82.1) |
| Number of psychiatric ED admissions*  1-year pre-initiation  1-year post-initiation | 0.17 (0.6)  0.12 (0.6) | 0.19 (0.7)  0.12 (0.6) | 0.16 (0.8)  0.13 (1.7) | 0.17 (0.6)  0.11 (0.6) | 0.18 (0.8)  0.13 (1.3) |

AP, antipsychotic; LAI, long-acting injectable antipsychotic; ED, emergency department; SD, standard deviation.

*All comparisons in the gender and age groups were significant (p<0.05) one year after versus one year before LAI initiation. Wilcoxon rank-sum test for paired samples.
